# Using the Behaviour Change Wheel Program Planning Model to Design Games for Health: Development Study

**DOI:** 10.2196/29964

**Published:** 2021-12-03

**Authors:** Michael C Robertson, Tom Baranowski, Debbe Thompson, Karen M Basen-Engquist, Maria Chang Swartz, Elizabeth J Lyons

**Affiliations:** 1 Department of Nutrition, Metabolism & Rehabilitation Sciences University of Texas Medical Branch at Galveston Galveston, TX United States; 2 U.S. Department of Agriculture/Agricultural Research Service Children’s Nutrition Research Center Baylor College of Medicine Houston, TX United States; 3 Department of Behavioral Science University of Texas MD Anderson Cancer Center Houston, TX United States; 4 Department of Pediatrics-Research University of Texas MD Anderson Cancer Center Houston, TX United States

**Keywords:** physical activity, video games, eHealth, intervention, behavior and behavior mechanisms, psychological theory, serious games, gamification, older women, older adults, behavior change, behavioral interventions, mobile phone

## Abstract

**Background:**

Games for health are a promising approach to health promotion. Their success depends on achieving both experiential (game) and instrumental (health) objectives. There is little to guide game for health (G4H) designers in integrating the science of behavior change with the art of game design.

**Objective:**

The aim of this study is to extend the Behaviour Change Wheel program planning model to develop Challenges for Healthy Aging: Leveraging Limits for Engaging Networked Game-Based Exercise (CHALLENGE), a G4H centered on increasing physical activity in insufficiently active older women.

**Methods:**

We present and apply the G4H Mechanics, Experiences, and Change (MECHA) process, which supplements the Behaviour Change Wheel program planning model. The additional steps are centered on identifying target G4H player experiences and corresponding game mechanics to help game designers integrate design elements and G4H objectives into behavioral interventions.

**Results:**

We identified a target behavior of increasing moderate-intensity walking among insufficiently active older women and key psychosocial determinants of this behavior from self-determination theory (eg, autonomy). We used MECHA to map these constructs to intervention functions (eg, persuasion) and G4H target player experiences (eg, captivation). Next, we identified behavior change techniques (eg, framing or reframing) and specific game mechanics (eg, transforming) to help realize intervention functions and elicit targeted player experiences.

**Conclusions:**

MECHA can help researchers map specific linkages between distal intervention objectives and more proximal game design mechanics in games for health. This can facilitate G4H program planning, evaluation, and clearer scientific communication.

## Introduction

### Background

Games for health (Gs4H) are a promising venue through which to increase motivation for, and enjoyment associated with, health-related behaviors. Its success is largely dependent on eliciting a playful, enjoyable, fun experience while simultaneously delivering efficacious behavior change techniques (BCTs) [1]. Gs4H seem to effect change in various contexts [2-5]. However, existing studies provide limited guidance for developers interested in designing novel Gs4H. Studies often have confusing or sparse discussions of their game design elements, and specific game mechanics are not often linked to theory-based behavior change mechanisms. This lack of clarity limits the accumulation of scientific knowledge and may be due in part to a lack of a common program planning method.

The extent to which a game for health (G4H) brings about behavior change defines its success. G4H developers have long recognized the potential utility of using insights from the field of behavioral science to achieve health-related behavior change [6]. BCTs have become a popular choice for parsing the *active ingredients* of behavioral interventions because they cut across diverse behavior change theories and help to explicate mechanisms of behavior change. Gs4H may be a particularly useful avenue through which to implement BCTs because they may be amenable to doing so with high fidelity to large numbers of people [7]. Explicitly including BCTs in G4H design will help G4H designers to identify the most effective strategies for fostering behavior change in various contexts and articulate hypothesized mechanisms of behavior change [7-9].

Detailed program planning methods exist for traditional health promotion programs. Systematic methods such as Intervention Mapping and the Behaviour Change Wheel (BCW) emphasize using behavior change theories and techniques to link modifiable determinants of behavior to health-related behaviors and outcomes [10-12]. However, these approaches do not readily extend to the intricacies of game design. For example, application of the BCW might indicate that the use of a BCT such as *framing* or *reframing* is warranted to influence health-related behavior [13], but researchers have little guidance on *how* to implement this BCT using game design. Research centered on using program planning methods in game design may facilitate more rigorous development and clear scientific communication of Gs4H.

Models of gamification or using game design elements to achieve nongame objectives emphasize the importance of designing game systems that are chiefly centered on the benefits to, and interests of, the user [14-16]. They highlight the importance of iterative prototyping and the potential utility of self-determination theory (SDT)—a health behavior change theory that posits that the satisfaction of one’s core psychological needs can have a bearing on one’s quality of motivation for engaging in health-related behavior [17,18]. The Mechanics, Dynamics, and Aesthetics Framework provides a formal approach to understanding games by helping designers to decompose games into their constituent design elements and map their interrelationships [18]. The Learning Mechanics-Game Mechanics Model enables researchers to map pedagogical principles featured in educational games to corresponding game design elements [19]. Existing models provide useful nuance but may not match the particular needs of behavioral health interventionists because they are not well suited for explicating mechanisms of behavior change.

Most Gs4H do not seem to be grounded in models of gamification or health behavior change theory [20]. Furthermore, although BCTs are commonly found in Gs4H (typically falling into the categories of feedback and monitoring, comparison of behavior, and reward and threat), their putative role in the experiences evoked by Gs4H are seldom specified [21]. Thus, at present there are not strong linkages between the core tenets of health behavior change theory and the experiential objectives of Gs4H (eg, fun or playfulness) [22]. This gap stymies progress in G4H design because it limits our ability to integrate key experiential and instrumental objectives and the precision with which we are able to evaluate the pathways through which Gs4H may effect change.

### Step-by-Step Program Planning Model

The development of Gs4H typically demands considerable up-front costs [9], and potential funders need to see how game design elements purport to effect health-related outcomes [23]. Thus, it is especially important to ensure that key scientific and game design principles are adequately integrated early in design. The BCW specifies commonly accepted BCTs and how to select them for program design [11,12]. We present a step-by-step program planning model adapting the BCW [11,12] to the development of Challenges for Healthy Aging: Leveraging Limits for Engaging Networked Game-Based Exercise (CHALLENGE). CHALLENGE is a social media game, delivered through Facebook and centered on increasing physical activity in insufficiently active older women. This paper thereby illustrates a process to guide researchers in the development of Gs4H and also provides a template to facilitate clearer scientific communication for linking behavior change objectives to G4H design.

## Methods

### Overview

**Figure 1:**
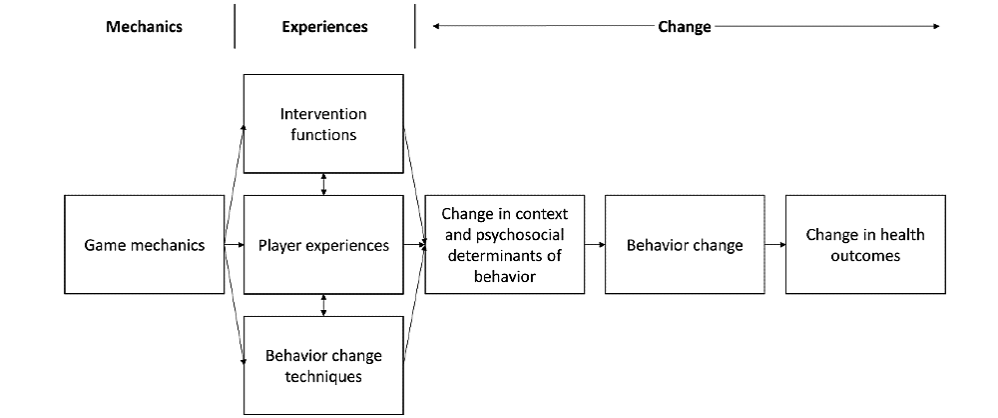
A Mechanics, Experiences, and Change Model for game-induced behavior change.

To develop the behavioral intervention core of CHALLENGE, we adapted the BCW program planning process enumerated by Michie et al [12] by adding 2 steps and removing 1. The first added step, termed *Identify target player experiences,* is centered on selecting target experiences that are conducive to intervention functions and influencing psychosocial constructs. The second added step, termed *Identify game mechanics*, is centered on selecting specific game design elements for eliciting target player experiences and implementing BCTs. We removed the BCW step centered on identifying policy categories because Gs4H are typically concerned with targeting intrapersonal- and interpersonal-level factors. Taken together, we call this G4H planning process the Mechanics, Experiences, and Change (MECHA) Model (Figure 1). It consists of 9 steps: (1) define the problem in behavioral terms, (2) select the target behavior, (3) specify the target behavior, (4) identify what needs to change, (5) identify intervention functions, (6) identify target player experiences, (7) select BCTs, (8) identify game mechanics, and (9) determine mode of delivery (Table 1).

### Study Approval

This study was approved by the institutional review board of The University of Texas Medical Branch at Galveston (protocol number: 19-0158).

**Table 1:** Steps of game for health Mechanics, Experiences, and Change Model.

Step	Description	Example
Step 1: Define the problem in behavioral terms^a^	Identify the specific target population. Review the epidemiological evidence concerning the health-related outcomes of interest. Identify relevant behaviors linked to these outcomes and their location in that target population	We identified the target population as women aged 65-85 years who are not meeting nationally recommended physical activity guidelines
Step 2: Select a target behavior^a^	Select a target behavior from the relevant behaviors identified in step 1. Consider the relative impact of each behavior, its likelihood of change, the potential for spillover into other important behaviors, and ease of measurement of the behavior	The nationally recommended physical activity guidelines for older adults include several related behaviors (eg, aerobic physical activity, muscle-strengthening physical activity, and time spent sedentary) [24]. In this step we opted to focus on aerobic physical activity because of its well-documented health benefits, preference in older adults [25], and amenability to objective measurement
Step 3: Specify the target behavior^a^	Specify the target behavior identified in step 2 in detail. To do so, use the 6 template questions proposed by Michie et al [12]: (1) Who needs to perform the behavior? (2) What does the person need to do differently to achieve the desired change? (3) When will they do it? (4) Where will they do it? (5) How often will they do it? (6) With whom will they do it?	We specified details pertaining to the target behavior selected in step 2 (presented in the *Results* section)
Step 4: Identify what needs to change^a^	Conduct a behavioral analysis as recommended by Michie et al [12] (ie, focus groups, questionnaires, observations, documentary analysis, or review of relevant literature) to identify psychosocial constructs that need to change to achieve the desired behavior change. Select an appropriate health-related behavior change theory or model to guide this process	We identified key constructs that need to change to promote the target behavior identified in step 3 (the *Results* section provides an illustrative template). This process may be guided by, for example, SDT^b^ [17,18] or the COM-B^c^ Model [12]. For example, *competence* for engaging in physical activity predicts physical activity levels across a wide range of contexts [26]
Step 5: Identify intervention functions^a^	Identify the primary intervention functions of the G4H^d^ intervention to target each of the constructs identified in step 4. The process of mapping intervention functions to psychosocial constructs can be guided by the BCW^e^ [12] and its subcomponents [27]	*Modeling*, for example, is an intervention function that may be used to increase feelings of competence for engaging in physical activity (a psychosocial construct featured in SDT)
Step 6. Identify target player experiences	Identify experiential objectives that would facilitate compelling gameplay and can be integrated with the intervention functions (identified in step 5). Behavior change theory can facilitate this process	We used the Playful Experiences Framework to frame the identification of target player experiences [28,29]. As an example, SDT suggests that experiences of friendly *competition* with appropriately matched role models may bolster feelings of *competence* for engaging in physical activity
Step 7: Identify BCTs^a,f^	Select the BCTs to be featured in the intervention. BCTs are the “active ingredients” of a behavioral intervention, or “the observable, replicable, irreducible components of an intervention” designed to change behavior (eg, the provision of feedback). The taxonomy of 93 distinct BCTs developed by Michie et al [13] can be used to frame the selection of BCTs. Review the extant scientific literature to identify BCTs that may be most effective in the context of the target behavior and target population	Existing literature suggests that prompting self-monitoring of behavior, as an example, may be particularly useful for helping older adults increase physical activity [30]
Step 8: Identify game mechanics	Identify specific game mechanics designed to evoke target participant experiences [15,31]. Select a taxonomy of game mechanics to guide G4H game design [32-37]. Map individual game mechanics to the player experiences, BCTs, and psychosocial constructs identified in previous steps. These conceptual links can be evaluated and refined during iterative game development [15,31]	As an example, participants’ physical activity performance (game mechanics) [33] may be used to evoke experiences of *competition* and implement the BCT of *social comparison*. These processes can be oriented toward increasing participants’ feelings of *competence* for engaging in physical activity
Step 9: Identify mode of delivery^a^	Identify modes of delivery of the intervention that would be appropriate for the target population. These decisions can be informed by formative research and the scientific literature	For example, previous research may suggest that older adults tend to prefer computer-based Gs4H to those delivered through mobile devices

^a^Michie et al [12] and Michie et al [13].

^b^SDT: self-determination theory.

^c^COM-B: Capability-Opportunity-Motivation Behavior.

^d^G4H: game for health.

^e^BCW: Behaviour Change Wheel.

^f^BCT: behavior change technique.

## Results

### Application of MECHA

We applied MECHA to develop CHALLENGE. In doing so, we sought to refine our understanding of the context of the behavior in the target population and mapped the hypothesized linkages between health behavior change theory constructs, intervention functions, target participant experiences, BCTs, and game mechanics.

### Step 1: Define the Problem in Behavioral Terms

Sustained moderate-to-vigorous intensity physical activity is beneficial for older adults [38]. Most older adults do not meet recommended levels of physical activity [39], and physical activity levels tend to decline with age [40,41]. Women tend to be less physically active than men [42-45] and can face decreased lean muscle mass after menopause [46] that can exacerbate the negative effects of inadequate activity [47]. Accordingly, the target population for this G4H includes being female, aged 65-85 years, owning a smartphone and having regular internet access, self-reported weekly moderate-to-vigorous intensity physical activity ≥150 minutes, and evidence suggesting that engaging in moderate-intensity physical activity would be safe (eg, answering *no* to all Physical Activity Readiness Questionnaire for Everyone items or having a physician’s note).

### Step 2: Select a Target Behavior

Moderate-intensity (ie, 3.0-6.0 metabolic equivalents) brisk walking has consistently emerged as a physical activity preference of older adults and can satisfy the nationally recommended aerobic physical activity guideline of engaging in at least 150 minutes of moderate to vigorous intensity physical activity per week [24,25]. Thus, we designed CHALLENGE to be primarily centered on increasing moderate-intensity walking.

### Step 3: Specify the Target Behavior

Keeping in mind the existing literature norms for older adults and findings from pilot study research [48], we selected a default step goal recommendation of walking at least 8000 steps per day for at least 5 days per week (Textbox 1).

**Specify the target behavior.**

**Specify the target behavior identified in detail by using the 6 template questions proposed by Michie et al [12]**

- Target behavior
- Walking at least 8000 steps per day for at least 5 days per week
- Who needs to perform the behavior?
- Women aged 65-85 years in southeast Texas who are not meeting nationally recommended aerobic physical activity guidelines
- What do they need to do differently to achieve the desired change?
- Increase walking, both as a lifestyle and for exercise. Increases should occur gradually with a target of ≥1000 daily steps per week until target goals are met
- When do they need to do it?
- Daily
- Where do they need to do it?
- Outdoors if possible, indoors at large venues, or at home
- How often do they need to do it?
- Daily
- With whom do they need to do it?
- Alone or in small groups

### Step 4: Identify What Needs to Change

We chose to ground the intervention in SDT [17,18] because it has led to better understanding of physical activity patterns [26] and effective game design [49,50]. We selected 2 constructs from SDT as the primary targets of CHALLENGE: intrinsic regulation and integrated regulation. Rather than focus on intrinsic regulation per se, we opted to focus on the basic psychological needs that are theorized to predict this form of motivation: perceived autonomy, competence, and relatedness. Although these psychological needs also predict integrated regulation, we focused on the construct separately to account for its emphasis on personal values and identity (Table 2).

**Table 2:** Identify what needs to change^a^.

Theoretical constructs	Requirements for the target behavior to occur	Should the intervention target this construct?
Perceived autonomy	Participant wants to engage in physical activity for autonomous reasons (ie, enjoyment, interest, identity, and values) [17,18,51]	Yes; autonomous motivations for physical activity predict long-term compliance to physical activity goals, and older women want autonomy-promoting interventions [26,52-54]
Perceived competence	Participant feels competent and able to engage in physical activity	Yes; self-efficacy is a strong predictor of physical activity, and many older women report low levels of self-efficacy for consistently meeting nationally recommended physical activity guidelines [55,56]
Perceived relatedness	Participant feels supported by others regarding her physical activity	Yes; social support is a strong predictor of physical activity, and older women want social physical activity interventions [55,57]
Intrinsic regulation	Participant perceives physical activity as fun and interesting	Yes; previous studies suggest that these factors predict physical activity, and this is an identified barrier in this population [54,55]
Integrated regulation	Participant perceives physical activity as being in line with her values and identity	Yes; previous studies suggest that integrated regulation predicts physical activity in this population [53-55,58]
Identified regulation	Participant perceives physical activity as associated with an outcome that is important to her	Yes; previous studies suggest that identified regulation predicts physical activity in this population [53-55,58]
Introjected regulation	Participant feels obligated to engage in physical activity	No; although in some cases this type of motivation may lead to behavior initiation, it is not conducive to long-term adherence to physical activity [26]
External regulation	Participant perceives physical activity as something that outside forces are encouraging her to do	No; although in some cases this type of motivation may lead to behavior initiation, it is not conducive to long-term adherence to physical activity [26]

^a^Target behavior: walking at least 8000 steps per day for at least 5 days per week.

### Step 5: Identify Intervention Functions

We identified 6 functions of our intervention to bring about change in the key psychological needs enumerated in Table 2. These were persuasion, incentivization, environmental restructuring, modeling, training, and enablement (Multimedia Appendix 1 [11-13,17,18,28,29,33]). We arrived upon these intervention functions by first reviewing the various intervention functions outlined by Michie et al [11,12]. We drew from this list of potential intervention functions in an iterative process as we proceeded through the remaining steps of MECHA to map our G4H intervention. In doing so, we paired specific intervention functions with specific target playful experiences to ensure that our intervention’s featured elements of game design would be integrated with these core tenets of behavior change.

### Step 6: Identify Target Player Experiences

We selected the Playful Experiences Framework to guide our identification of target player experiences [28,29]. This framework was derived from studies of electronic art and video games and was created to help designers make more engaging and attractive participative systems. Using this framework, we selected target experiences for bolstering participants’ psychological needs in accordance with SDT tenets (Multimedia Appendix 1). For example, because an individual’s sense of autonomy is largely derived from their feelings of volitional control and authentic endorsement of their own behavior [17,18], we sought to evoke sensations of captivation, discovery, exploration, expression, and humor in the course of their gameplay. Note that the connections we make between participant experiences and SDT constructs are not necessarily exclusive; they are those that we believe are the strongest links conceptually, but it is likely that player experiences could target multiple theoretical constructs.

### Step 7: Identify BCTs

We identified 13 BCTs to increase moderate-intensity walking in CHALLENGE by pairing the target player experiences identified in step 6 with the taxonomy of 93 distinct BCTs described by Michie et al [13] (Multimedia Appendix 1). These were as follows: adding objects to the environment, framing or reframing, goal setting for behavior, graded tasks, identity associated with changed behavior, information about others’ approval, nonspecific reward, prompts or cues, reducing negative emotions, self-monitoring of behavior, self-talk, social comparison, and social reward.

### Step 8: Identify Game Mechanics

We selected the library of game mechanics developed by Järvinen [33] to guide our intervention design (1) for its balance of complexity and parsimony and (2) because we felt that this conceptualization of game mechanics, defined as “what the player does in relation to the game state during a standard turn or sequence,” would lend itself to autonomy-supportive, meaningful game design. We identified game mechanics from this library to elicit targeted player experiences and implement BCTs (Multimedia Appendix 1). Note that these connections are not necessarily exclusive; the mapped connections are those that we believe are the strongest links conceptually, but it is likely that many of these elements of gameplay could engender varied player experiences.

### Step 9: Identify Mode of Delivery

We identified Facebook as an appropriate platform through which to deliver this intervention [59,60]. We also decided to provide participants with digital physical activity trackers. Other studies have shown high acceptability of these technologies for delivering physical activity interventions to insufficiently active older adults [61,62].

### Developed G4H

Table 3 presents details of the behavioral intervention using the Template for Intervention Description and Replication Checklist [63]. Participants receive weekly challenges for 1 year. Challenges are centered on implementing the game mechanics identified previously (Multimedia Appendix 1; example challenges). At baseline, participants receive materials that can be used for some challenges throughout their participation in the study (eg, description cards for scavenger hunt–style challenges and cardboard frames, as well as masks and similar props that allow participants to obscure their faces in photographs). Weekly challenges are delivered by trained moderators, who post in a private Facebook group (Table 3; moderators are research staff trained by the principal investigator [EJL] on basic aspects of the Playful Experiences Framework [28,29] and the intended mechanisms of change of the intervention). Participants are encouraged to comment in response to the weekly challenges and interact with each other in this forum at their convenience throughout the week. All challenges were presented to a sample of individuals from the target population (n=20) in individual interviews, and we conducted an in-depth, qualitative evaluation of the interview transcripts and revised the intervention content according to participant feedback (manuscript in preparation).

**Table 3:** Template for Intervention Description and Replication Checklist for Challenges for Healthy Aging: Leveraging Limits for Engaging Networked Game-Based Exercise (CHALLENGE).

Item name	Item
Brief name	CHALLENGE
Why	Despite short-term benefits, older adults’ adherence to physical activity and tracker use decrease sharply over time. Most existing intervention systems use a corrective frame: they are oriented toward *fixing* undesirable behaviors. Emerging theoretical frameworks indicate that this approach is unlikely to produce sustained behavior change. Instead, taking a celebratory approach that facilitates enjoyable and valued aspects of behavior may better promote longer-term adherence to physical activity. Games for health are conducive to this approach to physical activity promotion
What (materials)	Participants are provided a wrist-worn electronic physical activity tracker (Fitbit Inspire 2 [Google LLC]) and various props (eg, scavenger hunt bingo cards and sunglasses or masks to help obscure their identities). If participants do not already have Facebook and Fitbit accounts, study staff help the participants to create them
What (procedures)	Participants meet face-to-face with study staff for orientation procedures (eg, aiding with technology) and data collection. Participants engage in goal setting and action planning with study staff at baseline and are invited to join a private Facebook group. Through this private Facebook group, participants receive weekly challenges that are centered on encouraging walking behaviors and eliciting playful experiences (see examples in Multimedia Appendix 1). Participants are encouraged to directly respond to challenges through Facebook posts and like or comment on others’ posts. Participants also receive weekly messages providing feedback on their physical activity levels and study engagement (ie, number of times participants posted in the Facebook group)
Who provided	Interventionists are trained by the principal investigator (EJL) on basic aspects of the Playful Experiences Framework [28,29] and the intended mechanisms of change of the intervention. Moderators are also trained by a key collaborator (MCS) who oversaw formative research for this intervention
How	Goal setting and action planning are conducted face-to-face or through videoconferencing at the start of the study. All other intervention content is delivered on the web. Challenges are posted weekly using social media to a single, private Facebook group. Participants also receive individual weekly emails presenting their device-measured physical activity levels, suggested goals, and engagement level
Where	Face-to-face meetings and data collection sessions are held at a large medical research university in southeast Texas. The intervention content is largely delivered through the internet
When and how much	Intervention content is sent weekly over the course of 1 year for participants, with participants being enrolled on a rolling basis until the target sample size is reached (estimated to be 2-3 years). Recruitment began in June 2021
Tailoring	At the beginning of the intervention, study staff meet with participants to establish physical activity goals (ie, target step count and number of days per week that participants aim to meet that target step count). In this meeting, participants also select their target weekly improvement rate (eg, participants may indicate that if they did not meet their target step count one week, then they would like their goal for the next week to be to increase their daily average step count by 1000). Weekly emails accordingly present feedback on the previous week and provide a suggested step count goal for the upcoming week
How well (planned)	Moderators’ weekly posts are based on a set schedule and accompanying scripts. The only communication with participants that is not heavily based on scripts are responses to direct messages or SMS text messages sent regarding scheduling, reporting unacceptable content, and so on. We will extract information from the Facebook group and Fitbit app regularly to track participant engagement

Human involvement in the G4H intervention is conducted by moderators, who facilitate all web-based proceedings. They post all weekly challenges with example responses, review the Facebook group for adverse events or inappropriate comments, provide supportive and clarifying comments to study participants as necessary, and publicly recognize consistent participation (eg, badges awarded to *power users*). Moderators also send weekly emails to participants with basic weekly feedback on their device-measured step count data. Trained interventionists perform face-to-face follow-up data collection procedures at 6, 12 (intervention end), and 18 months after baseline. After intervention end, participants can elect to stay in the Facebook group if they wish (to facilitate an active, supportive environment). Participants are compensated for their time with a US $25 gift card at each of the 3 follow-up appointments.

To evaluate the G4H, we aim to recruit 300 participants reflective of the target population detailed in step 1. We will recruit participants on a rolling basis by using several strategies, including in-person recruitment at gerontology and primary care clinics, web-based recruitment methods, and flyers and brochures placed at locations frequented by members of the target population. Outcome measures for the developed G4H include objectively measured step count and moderate to vigorous physical activity levels [64], as well as measures of physical functioning (eg, 6-minute walk test [65,66]). We will also investigate potentially mediating variables (eg, the psychosocial constructs presented in Table 2 [67-69]), the role of process variables such as those reflecting participant engagement (eg, participants’ weekly number of posts, comments, reactions, days engaged, and total number of engagements), and the degree to which the experimental group, compared with the comparison group, experienced the targeted playful experiences over the course of the study period [29]. We will investigate predictors of participant engagement (eg, age and fitness level) and how engagement is associated with study outcomes.

## Discussion

### Principal Findings

We extended the BCW to create MECHA, a step-by-step program planning model for designing Gs4H, and applied it to the development of a behavioral intervention centered on increasing physical activity in insufficiently active older women. MECHA may help researchers map specific linkages between distal instrumental and experiential objectives to the more proximal elements of game design. This may facilitate program planning, evaluation, and clearer communication of results to expedite scientific accumulation of knowledge.

### Comparison With Prior Work

MECHA shares similarities with other models that can help researchers parse G4H game design elements. The Mechanics, Dynamics, and Aesthetics Framework emphasizes the importance of mapping game mechanics to targeted emotional responses in game design [31]. This is analogous to the process of mapping game mechanics to targeted player experiences presented in this study. The Mechanics, Dynamics, and Aesthetics Framework can supplement this process for G4H designers. The Learning Mechanics-Game Mechanics Model [19] is centered on mapping game mechanics to specific educational objectives when designing games for educational purposes. This is somewhat analogous to the process of mapping game mechanics to BCTs that we encourage in this study. These models and the research derived from them underscore the utility of mapping game mechanics to desired objectives for intervention development and evaluation.

We used the library of primary game mechanics developed by Järvinen [33], but other taxonomies of game mechanics may be useful for different Gs4H [32-37]. Game mechanics interact with one another and may have different effects at different times and different effects on different users; because of this inherent complexity, it is unlikely that a single, definitive catalog of game mechanics will emerge [32,70]. The conceptualization of game mechanics described by Järvinen [33] comports with the theoretical underpinnings and context of our G4H. Other taxonomies range from the conceptualization of game mechanics described by Schell [32] as consisting of the 7 essential elements of space, time, objects, actions, rules, skill, and chance to the extensive list of hundreds of game design patterns compiled by Bjork and Holopainen [71]. Designers should select from among existing taxonomies of game mechanics the one that provides the greatest utility for integrating the results of the preceding steps presented in this paper.

Evoking enjoyable participant experiences is likely critical to sustained adherence to Gs4H and their associated health-related behaviors but designing games that do so reliably is a challenge. Consumption of games, as opposed to consumption of books, movies, and so on, is inherently nonlinear, and this introduces some uncertainty in predicting player experience [31,32]. A key to honing the desired qualities of emergent gameplay is iterative development with frequent input from the priority population [15,31]. Semistructured qualitative interviews with participants who experience the G4H can be a useful tool for investigating to what degree participants are indeed undergoing the targeted experiences. Quantitative data may further help researchers divine participants’ experiences. The Playful Experiences Questionnaire, for example, measures the categories promulgated in the Playful Experiences Framework [28,29]. Research with mixed methods designs that allow qualitative and quantitative data to build upon one another may lead to a deeper level of understanding.

We identified areas of needed research while conducting this project. First, more research is needed to elucidate how game experiences may affect SDT constructs. Greater clarity regarding if and how different experiences afforded by Gs4H affect critical psychosocial constructs may help game designers to develop more efficacious Gs4H. Second, more research that explicates how different G4H game mechanics engender specific player experiences is needed. Although this is likely to remain the purview of experts because of its inherent complexity, literature that helps to frame these links may help researchers to systematize iterative game development and communicate research proposals and study findings. Third, engagement is a key issue in securing a person’s participation in, and thereby exposure to, a game. Thus, engagement is critical for G4H effectiveness. Engagement likely hinges on participants’ G4H-related experiences—both experiences stemming from the game and the health-promoting aspects of the G4H. More research is needed to characterize how specific G4H-related experiences correspond to effective engagement with the G4H [72,73].

### Strengths and Limitations

The strengths of this study include adherence to recommended procedures for increasing transparency of scientific research (eg, the Template for Intervention Description and Replication Checklist), a systematic approach to game design, and the real-world application of the G4H development process to create a behavioral intervention. The limitations of this study include, first, that we did not conduct a formal systematic review of the literature. As G4H design exists at the confluence of several fields, this was not within the scope of this study. Although our study team has considerable expertise in G4H research, there may be additional relevant studies not covered in this paper. Furthermore, our study team did not include professional game designers or developers. We made extensive use of relevant scientific literature, but future research would benefit from the inclusion of individuals with expertise in these areas. The MECHA model is a useful starting point, but it would likely be improved by incorporating the unique insights that game designers and experts in human-computer interaction may provide. This may be especially useful as MECHA is applied to help design health-promoting video games.

### Conclusions

G4H design combines the art of game design and the science of behavior change. In this paper, we have presented a process for systematically integrating these perspectives and illustrated its use in the design of a G4H centered on increasing physical activity in insufficiently active older adults. This systematic approach to G4H design may facilitate program planning, evaluation, and clearer communication of G4H interventions.

## Appendix

Multimedia Appendix 1 Mapping self-determination theory constructs, intervention functions, target participant experiences, behavior change techniques, and game mechanics.

## Abbreviations

BCT: behavior change technique
CHALLENGE: Challenges for Healthy Aging: Leveraging Limits for Engaging Networked
G4H: game for health
Gs4H: games for health
MECHA: Mechanics, Experiences, and Change
SDT: self-determination theory

## References

[1] Baranowski MT, Lieberman PD, Buday R, Peng W, Zimmerli L, Wiederhold B, Kato PM (2013). Videogame mechanics in games for health. Games Health J.

[2] Papastergiou M (2009). Exploring the potential of computer and video games for health and physical education: a literature review. Comput Educ.

[3] Lyons EJ, Tate DF, Ward DS, Ribisl KM, Bowling JM, Kalyanaraman S (2014). Engagement, enjoyment, and energy expenditure during active video game play. Health Psychol.

[4] Kappen DL, Mirza-Babaei P, Nacke LE (2018). Older adults’ physical activity and exergames: a systematic review. Int J Human Comput Interac.

[5] Epstein DS, Zemski A, Enticott J, Barton C (2021). Tabletop board game elements and gamification interventions for health behavior change: realist review and proposal of a game design framework. JMIR Serious Games.

[6] Thompson D, Baranowski T, Buday R, Baranowski J, Thompson V, Jago R, Griffith MJ (2010). Serious video games for health how behavioral science guided the development of a serious video game. Simul Gaming.

[7] Baranowski T, Thompson D, Buday R, Lu AS, Baranowski J (2010). Design of video games for children's diet and physical activity behavior change. Int J Comput Sci Sport.

[8] Baranowski T, Baranowski J, Thompson D, Buday R (2011). Behavioral science in video games for children's diet and physical activity change: key research needs. J Diabetes Sci Technol.

[9] Baranowski T, Buday R, Thompson D, Lyons EJ, Lu AS, Baranowski J (2013). Developing games for health behavior change: getting started. Games Health J.

[10] Bartholomew L, Parcel G, Kok G, Gottlieb N, Schaalma H, Markham C, Tyrrell S, Shegog R, Fernandez M, Mullen P, Gonzalez A (2006). Planning Health Promotion Programs: An Intervention Mapping Approach.

[11] Michie S, van Stralen MM, West R (2011). The behaviour change wheel: a new method for characterising and designing behaviour change interventions. Implement Sci.

[12] Michie S, Atkins L, Gainforth H (2016). Changing behaviour to improve clinical practice and policy. Novos Desafios Novas Competências Contrib Atuais Psicol Braga Axioma-Publicações Fac Filos.

[13] Michie S, Richardson M, Johnston M, Abraham C, Francis J, Hardeman W, Eccles MP, Cane J, Wood CE (2013). The behavior change technique taxonomy (v1) of 93 hierarchically clustered techniques: building an international consensus for the reporting of behavior change interventions. Ann Behav Med.

[14] Nicholson S (2015). A recipe for meaningful gamification. Gamification in Education and Business.

[15] Deterding S (2015). The lens of intrinsic skill atoms: a method for gameful design. Human Comput Interac.

[16] Verschueren S, Buffel C, Vander Stichele G (2019). Developing theory-driven, evidence-based serious games for health: framework based on research community insights. JMIR Serious Games.

[17] Ryan RM, Deci EL (2000). Self-determination theory and the facilitation of intrinsic motivation, social development, and well-being. Am Psychol.

[18] Deci EL, Ryan RM (2008). Self-determination theory: a macrotheory of human motivation, development, and health. Can Psychol/Psychologie canadienne.

[19] Arnab S, Lim T, Carvalho M, Bellotti F, Freitas S, Louchart S, Suttie N, Berta R, De Gloria A (2015). Mapping learning and game mechanics for serious games analysis. Br J Educ Technol.

[20] Lu AS, Kharrazi H (2018). A state-of-the-art systematic content analysis of games for health. Games Health J.

[21] Edwards EA, Lumsden J, Rivas C, Steed L, Edwards LA, Thiyagarajan A, Sohanpal R, Caton H, Griffiths CJ, Munafò MR, Taylor S, Walton RT (2016). Gamification for health promotion: systematic review of behaviour change techniques in smartphone apps. BMJ Open.

[22] Cugelman B (2013). Gamification: what it is and why it matters to digital health behavior change developers. JMIR Serious Games.

[23] Baranowski T (2018). Games for health research—past, present, and future. Präv Gesundheitsf.

[24] Piercy KL, Troiano RP, Ballard RM, Carlson SA, Fulton JE, Galuska DA, George SM, Olson RD (2018). The physical activity guidelines for Americans. JAMA.

[25] Amireault S, Baier JM, Spencer JR (2018). Physical activity preferences among older adults: a systematic review. J Aging Phys Act.

[26] Teixeira PJ, Carraça EV, Markland D, Silva MN, Ryan RM (2012). Exercise, physical activity, and self-determination theory: a systematic review. Int J Behav Nutr Phys Act.

[27] Cane J, O'Connor D, Michie S (2012). Validation of the theoretical domains framework for use in behaviour change and implementation research. Implement Sci.

[28] Korhonen H, Montola M, Arrasvuori J (2009). Understanding playful user experience through digital games. Proceedings of the International Conference on Designing Pleasurable Products and Interfaces.

[29] Boberg M, Karapanos E, Holopainen J, Lucero A (2015). PLEX-Q: towards a playful experiences questionnaire. Proceedings of the 2015 Annual Symposium on Computer-Human Interaction in Play.

[30] French DP, Olander EK, Chisholm A, Mc Sharry J (2014). Which behaviour change techniques are most effective at increasing older adults' self-efficacy and physical activity behaviour? A systematic review. Ann Behav Med.

[31] Hunicke R, LeBlanc M, Zubek R (2004). MDA: a formal approach to game design and game research. Proceedings of the AAAI Workshop on Challenges in Game AI.

[32] Schell J (2020). The Art of Game Design: A Book of Lenses. Third edition.

[33] Järvinen A (2008). Games Without Frontiers: Methods for Game Studies and Design.

[34] Adams E, Dormans J (2012). Game Mechanics: Advanced Game Design.

[35] Hervas R, Ruiz-Carrasco D, Mondejar T, Bravo J (2017). Gamification mechanics for behavioral change: a systematic review and proposed taxonomy. Proceedings of the 11th EAI International Conference on Pervasive Computing Technologies for Healthcare.

[36] Toda A, Oliveira W, Klock A, Palomino P, Pimenta M, Gasparini I, Shi L, Bittencourt S, Isotani S, Cristea A (2019). A taxonomy of game elements for gamification in educational contexts: proposal and evaluation. Proceedings of the IEEE 19th International Conference on Advanced Learning Technologies (ICALT).

[37] Cechetti N, Biduki D, De Marchi A (2017). Gamification strategies for mobile device applications: a systematic review. Proceedings of the 12th Iberian Conference on Information Systems and Technologies (CISTI).

[38] Taylor A, Cable N, Faulkner G, Hillsdon M, Narici M, Van Der Bij A (2004). Physical activity and older adults: a review of health benefits and the effectiveness of interventions. J Sports Sci.

[39] Jefferis BJ, Sartini C, Lee I, Choi M, Amuzu A, Gutierrez C, Casas JP, Ash S, Lennnon LT, Wannamethee SG, Whincup PH (2014). Adherence to physical activity guidelines in older adults, using objectively measured physical activity in a population-based study. BMC Public Health.

[40] Evenson K, Buchner D, Morland K (2012). Objective measurement of physical activity and sedentary behavior among US adults aged 60 years or older. Prev Chronic Dis.

[41] Clarke CL, Sniehotta FF, Vadiveloo T, Argo IS, Donnan PT, McMurdo ME, Witham MD (2017). Factors associated with change in objectively measured physical activity in older people - data from the physical activity cohort Scotland study. BMC Geriatr.

[42] Gretebeck KA, Sabatini LM, Black DR, Gretebeck RJ (2017). Physical activity, functional ability, and obesity in older adults: a gender difference. J Gerontol Nurs.

[43] Aoyagi Y, Shephard RJ (2013). Sex differences in relationships between habitual physical activity and health in the elderly: practical implications for epidemiologists based on pedometer/accelerometer data from the Nakanojo Study. Arch Gerontol Geriatr.

[44] Straight C, Brady A, Evans E (2015). Sex-specific relationships of physical activity, body composition, and muscle quality with lower-extremity physical function in older men and women. Menopause.

[45] Brugnara L, Murillo S, Novials A, Rojo-Martínez G, Soriguer F, Goday A, Calle-Pascual A, Castaño L, Gaztambide S, Valdés S, Franch J, Castell C, Vendrell J, Casamitjana R, Bosch-Comas A, Bordiú E, Carmena R, Catalá M, Delgado E, Girbés J, López-Alba A, Martínez-Larrad MT, Menéndez E, Mora-Peces I, Pascual-Manich G, Serrano-Ríos M, Gomis R, Ortega E (2016). Low physical activity and its association with diabetes and other cardiovascular risk factors: a nationwide, population-based study. PLoS One.

[46] Hodson L, Harnden K, Banerjee R, Real B, Marinou K, Karpe F, Fielding BA (2014). Lower resting and total energy expenditure in postmenopausal compared with premenopausal women matched for abdominal obesity. J Nutr Sci.

[47] Ward-Ritacco C, Adrian A, Johnson M, Rogers L, Evans E (2014). Adiposity, physical activity, and muscle quality are independently related to physical function performance in middle-aged postmenopausal women. Menopause.

[48] Tudor-Locke C, Craig CL, Aoyagi Y, Bell RC, Croteau KA, De BI, Ewald B, Gardner AW, Hatano Y, Lutes LD, Matsudo SM, Ramirez-Marrero FA, Rogers LQ, Rowe DA, Schmidt MD, Tully MA, Blair SN (2011). How many steps/day are enough? For older adults and special populations. Int J Behav Nutr Phys Act.

[49] Przybylski AK, Rigby CS, Ryan RM (2010). A motivational model of video game engagement. Rev General Psychol.

[50] Ryan RM, Rigby CS, Przybylski A (2006). The motivational pull of video games: a self-determination theory approach. Motiv Emot.

[51] Caldwell AE, Masters KS, Peters JC, Bryan AD, Grigsby J, Hooker SA, Wyatt HR, Hill JO (2018). Harnessing centred identity transformation to reduce executive function burden for maintenance of health behaviour change: the Maintain IT model. Health Psychol Rev.

[52] Dacey M, Baltzell A, Zaichkowsky L (2008). Older adults' intrinsic and extrinsic motivation toward physical activity. Am J Health Behav.

[53] Janssen SL, Stube JE (2014). Older adults' perceptions of physical activity: a qualitative study. Occup Ther Int.

[54] Maula A, LaFond N, Orton E, Iliffe S, Audsley S, Vedhara K, Kendrick D (2019). Use it or lose it: a qualitative study of the maintenance of physical activity in older adults. BMC Geriatr.

[55] Kosteli M, Williams SE, Cumming J (2016). Investigating the psychosocial determinants of physical activity in older adults: a qualitative approach. Psychol Health.

[56] McAuley E, Szabo A, Gothe N, Olson EA (2011). Self-efficacy: implications for physical activity, function, and functional limitations in older adults. Am J Lifestyle Med.

[57] Lindsay Smith G, Banting L, Eime R, O'Sullivan G, van Uffelen JG (2017). The association between social support and physical activity in older adults: a systematic review. Int J Behav Nutr Phys Act.

[58] Jancey JM, Clarke A, Howat P, Maycock B, Lee AH (2009). Perceptions of physical activity by older adults: a qualitative study. Health Educ J.

[59] (2017). Tech adoption climbs among older Americans. Pew Research Center.

[60] (2021). Social media demographics to inform your brand's strategy in 2021. Sprout Social.

[61] Lyons EJ, Swartz MC, Lewis ZH, Martinez E, Jennings K (2017). Feasibility and acceptability of a wearable technology physical activity intervention with telephone counseling for mid-aged and older adults: a randomized controlled pilot trial. JMIR Mhealth Uhealth.

[62] Sinclair TJ, Grieve R (2017). Facebook as a source of social connectedness in older adults. Comput Human Behav.

[63] Hoffmann TC, Glasziou PP, Boutron I, Milne R, Perera R, Moher D, Altman DG, Barbour V, Macdonald H, Johnston M, Lamb SE, Dixon-Woods M, McCulloch P, Wyatt JC, Chan A, Michie S (2014). Better reporting of interventions: template for intervention description and replication (TIDieR) checklist and guide. BMJ.

[64] Tudor-Locke C, Camhi S, Troiano R (2012). A catalog of rules, variables, and definitions applied to accelerometer data in the National Health and Nutrition Examination Survey, 2003-2006. Prev Chronic Dis.

[65] Rikli R, Jones J (2013). Senior Fitness Test Manual.

[66] Ross RM, Murthy JN, Wollak ID, Jackson AS (2010). The six minute walk test accurately estimates mean peak oxygen uptake. BMC Pulm Med.

[67] Markland D, Tobin V (2004). A modification to the behavioural regulation in exercise questionnaire to include an assessment of amotivation. J Sport Exerc Psychol.

[68] Wilson P, Rodgers W, Loitz C, Scime G (2006). “It's Who I Am … Really!’ The importance of integrated regulation in exercise contexts. J Appl Biobehav Res.

[69] Vlachopoulos SP (2008). The basic psychological needs in exercise scale: measurement invariance over gender. Struct Equ Modeling.

[70] Sicart M (2008). Defining game mechanics. Game Stud Int J Comput Game Res.

[71] Björk S, Holopainen J (2006). Games and design patterns. Game Des Read.

[72] Yardley L, Spring BJ, Riper H, Morrison LG, Crane DH, Curtis K, Merchant GC, Naughton F, Blandford A (2016). Understanding and promoting effective engagement with digital behavior change interventions. Am J Prev Med.

[73] Michie S, Yardley L, West R, Patrick K, Greaves F (2017). Developing and evaluating digital interventions to promote behavior change in health and health care: recommendations resulting from an international workshop. J Med Internet Res.

