# Supplementary material for: Using the Behaviour Change Wheel Program Planning Model to Design Games for Health: Development Study
Source: JMIR Serious Games. 2021 Dec 3;9(4):e29964. doi: 10.2196/29964 (PMC8686484; doi:10.2196/29964)
Supplement: Multimedia Appendix 1 [file games_v9i4e29964_app1.docx]

**Multimedia Appendix 1.** Mapping self-determination theory constructs, intervention functions, target participant experiences, behavior change techniques, and game mechanics.

| SDT construct [17,18] (step 4) | Intervention function [11,12] (step 5) | Participant experiences [29,30] (step 6) | BCT [13] (step 7) | Game mechanic [33] (step 8) | Implementation in CHALLENGE^a^ (example) |
| --- | --- | --- | --- | --- | --- |
| **Autonomy** | | | | | |
|  | Persuasion (using communication to induce positive or negative feelings or stimulate action) | Captivation (losing one’s sense of time or forgetting one’s surroundings) | Framing or reframing (adoption of a perspective or new perspective on behavior to change cognitions and emotions about performing the behavior) | Transforming (the players are given an ability to transform the flow of time or space to better their chances of overcoming a challenge or to find out an outcome of their actions) | Being physically active is reframed as an action that affords progress in a game; challenges encourage seeing beauty in everyday things because participants pay particularly close attention to their environments during physical activity (“Change perspective. On your walks this week, pay close attention to the view from different places. How does it change? Do some points of view or times of day make for better views? Show us the best views that you can find.”) |
|  | Environmental restructuring (changing the physical or social context) | Discovery (finding something new or unknown) | Adding objects to the environment (add objects to the environment to facilitate performance of the behavior) | Information seeking (gathering information or making inquiries about surroundings, challenges, or other players) | Participants are provided with props that are featured in various challenges that encourage physical activity (“Scavenger hunt! Let’s play bingo with things you might find on your walks this week. We’ve given you a bingo card with two sides, one a little harder and one a little easier. See if you can get a bingo [or even a blackout!] and show us what you found.”) |
|  | Environmental restructuring (Changing the physical or social context) | Exploration (experience of exploring or investigating a world, affordance, puzzle, or situation) | Prompts or cues (introduce or define environmental or social stimulus with the purpose of prompting or cueing the behavior) | Browsing (browsing or moving through possible choices or instances of game elements) | Weekly challenges encourage exploring one’s surroundings in novel ways and finding out more on various topics (“Tour guide. Our area has lots of tours: historic homes, ghosts, even chicken coops! This week, look for new tour spots in your area. Show us some of the best stops on your tour and tell us why you chose them.”) |
|  | Persuasion (using communication to induce positive or negative feelings or stimulate action) | Expression (manifesting oneself creatively) | Identity associated with changed behavior (the person constructs a new self-identity) | Expressing (expressing oneself verbally with the means that the game system and technology affords); storytelling (telling or creating a story with the means that the game system affords and within its rules) | Participants are encouraged to creatively express themselves through various types of media in the context of solving challenges associated with physical activity (“Memento. Mementos are keepsakes that remind you of a person or a thing that happened. This week on your walks, take a picture as a memento of your day. What really made an impression on you? Take notice of how these photos change each day, and tell us all about it.”) |
|  | Enablement (increasing means or reducing barriers to increase capability or opportunity) | Humor (fun, joy, amusement, jokes, and gags) | Reduce negative emotions (reducing negative emotions to facilitate performance of the behavior) | Composing (the players are afforded means to create images and sounds) | The intervention encourages crafting casual, positive, fun, and sometimes silly responses to challenge (“Smile finder. Let’s spend this week finding things that make you smile or laugh. What brightened your day or gave you a good chuckle on your walks this week? Show us a picture and tell us all about it!”) |
|  | Enablement (increasing means or reducing barriers to increase capability or opportunity) | Relaxation (experience of unwinding, relaxation, or stress relief; calmness during play) | Reduce negative emotions (reducing negative emotions to facilitate performance of the behavior) | Submitting (submitting information [in a format specified in the rules] for evaluation by the game system or other players) | Participants share information with one another on subject matter that has no real-world impact; players can work together to complete goals with a sense of playfulness (—^b^) |
| **Competence** | | | | | |
|  | Training (imparting skills) | Challenge (testing abilities in a demanding task) | Goal setting for behavior (set or agree on a goal defined in terms of the behavior to be achieved); graded tasks (set easy-to-perform tasks, making them increasingly difficult but achievable until behavior is performed); self-monitoring of behavior (establish a method for the person to monitor and record their behaviors as part of a behavior change strategy) | Moving (players are allowed to physically move within the game environment); operating (taking an action where an object belonging to the game system [a component or the environment] is operated; usually the operation executes a game system procedure that produces information or change in other game element) | Participants are provided with a digital physical activity tracker. Step goals are discussed with participants, with an initial suggestion of approximately 3000 steps per day above baseline levels on 3 goal days per week and steady weekly increases until participants reach at least 8000 steps per day on 5 days per week. While making progress on step count goals, participants build knowledge of local history and nature and skills related to mindful attention, observation, and creativity. (“March 2nd is Texas Independence Day! This week, we’re looking at Texas state symbols. While you’re walking, look for the state bird (mockingbird), or state tree (pecan), or state fabric (cotton)! There are lots of other state symbols, so we’ve given you a list. See how many you can find!”) |
|  | Modeling (providing an example for people to aspire to or imitate) | Competition (contest with oneself or an opponent) | Social comparison (draw attention to others’ performance to allow comparison with the person’s own performance) | Performing (display of physical skill or physical performance, including simulations of physical performance, which is evaluated by the game system) | Participants engage in low-stakes, informal competitions with one another to overcome arbitrary challenges (“Count your chickens! This week, we’re having a competition to see who can find the most total birds over the week. While you’re walking, see how many birds you can count – flying, walking, on a sign, in a picture, in a cloud, lawn flamingos...Show us some of your bird encounters, and let us know your total at the end of the week. Chickens count double!”) |
|  | Incentivization (providing the expectation of reward) | Completion (finishing a major task, closure) | Nonspecific reward (delivery of a reward if and only if there has been effort and progress in performing the behavior) | Taking (taking a game element or a number of them [components, environment locations, or information] into possession) | Participants collect internet-based badges that represent their completion of each challenge (—) |
| **Relatedness** | | | | | |
|  | Persuasion (using communication to induce positive or negative feelings or stimulate action) | Fellowship (friendship, communality, or intimacy) | Social reward (verbal or nonverbal reward if and only if there has been effort or progress in performing the behavior) | Contracting (a contract by 2 or more players is made through an agreement that is acknowledged by the game system [ie, informal cooperation is formalized into a mechanic that makes the contract known to the game system]) | Participants *like* and comment on one another’s posts and learn about others from their photos and text (—) |
|  | Enablement (increasing means or reducing barriers to increase capability or opportunity) | Nurture (taking care of oneself or others) | Information about others’ approval (provide information about what other people think about the behavior. The information clarifies whether others will like, approve, or disapprove of what the person is doing or will do); self-talk (prompt positive self-talk [aloud or silently] before and during the behavior) | Conversing (players are able to enter into dialog with game system or other players, and this dialog has formal consequences for the game state) | Moderators provide encouraging comments in the Facebook group and encourage participants to validate others’ entries. Participants gain insight into their and others’ values and contribute to a supportive and caring environment (“Socialite. Let’s be social this week and think about our friends in the group. Can you find and take pictures of things that you think will make the other group members happy?”) |

^a^CHALLENGE: Challenges for Healthy Aging: Leveraging Limits for Engaging Networked Game-Based Exercise.

^b^Not available.
